# Development of an item pool for a patient reported outcome measure of resilience for people living with dementia

**DOI:** 10.1186/s41687-023-00638-z

**Published:** 2023-09-27

**Authors:** Jennifer Rhiannon Roberts, Catherine Anne MacLeod, Zoe Hoare, Mary Pat Sullivan, Emilie Brotherhood, Joshua Stott, Gill Windle

**Affiliations:** 1 School of Medical and Health Sciences, Bangor University, Bangor, UK; 2 Faculty of Education and Professional Studies, School of Social Work, Nipissing University, North Bay, ON Canada; 3 Dementia Research Centre, Queen Square Institute of Neurology, University College London (UCL), London, UK; 4 Department of Clinical, Educational and Health Psychology, University College London (UCL), London, UK

**Keywords:** Dementia, Resilience, Strengths-based, Measure

## Abstract

**Background and objectives:**

Policies to support people living with dementia increasingly focus on strengths-based approaches, highlighting the importance of building resilience. This research responds to the lack of a suitable resilience measure for people with dementia. It develops a pool of items to inform a new measure of resilience for this population.

**Methods:**

A conceptual model and associated data informed the item generation of the draft resilience measure. Regular meetings with professionals (n = 7) discussed response-scale formatting, content and face validity, leading to refinement and item reduction. Cognitive interviews with people living with dementia (n = 11) then examined the face and content validity of items and the suitability of response-scale formatting. These two phases informed subsequent revision and further item reduction of the resilience measure.

**Results:**

The first item generation exercise led to 140 items. These were independently assessed by the professionals and this refinement reduced the measure to 63 items across 7 domains of the conceptual model (psychological strengths; practical approaches for adapting to life with dementia; continuing with hobbies, interests and activities; strong relationships with family and friends; peer support and education; participating in community activities; the role of professional support services). Cognitive interviews explored the 63 items with people living with dementia. Detailed feedback led to items removed due to difficulty with (a) understanding (N = 7); (b) answering (n = 11); (c) low preference for that item (n = 6); and (d) presence of a preferred item within a cluster of similar questions (n = 4). Items were amended to enhance clarity/conciseness (n = 19) leading to a final 37-item pool.

**Conclusion:**

Established methods for measurement development included the expertise of people with dementia and led to the generation of a set of items for a new resilience measure that were understandable and acceptable to this target population. This 37-item pool reflects the conceptual understanding of resilience in dementia as being derived across individual, community and societal level resources.

**Supplementary Information:**

The online version contains supplementary material available at 10.1186/s41687-023-00638-z.

## Introduction

Dementia is a major international public health concern [1]. In the absence of readily available pharmacological therapies, enabling people to ‘live well’ with dementia remains a policy and practice priority [2]. In the UK (United Kingdom), legislation promotes well-being and independence through strengths-based approaches to supporting people requiring any level of care or support [3]. Resilience is a concept that focuses on strengths rather than deficits and is associated with ‘living well’ with dementia [4].

Historically resilience has been referred to as the ability to ‘bounce back’ [5] or to ‘positively adapt’ [6] in the face of adversity. Over time the concept has developed from being perceived as an individual trait, into a multidimensional construct incorporating individual traits, factors external to the individual, and how they interact. The ecological resilience framework and the WHO European policy framework suggest that resilience can be achieved by drawing on individual, community and societal assets [7, 8]. The literature around resilience in people living with dementia is sparse but gaining momentum. Windle et al. [9] suggest that the characterisation of resilience as ‘bouncing back’ may not capture the experience of people with dementia, who instead describe resilience as “managing and adapting under pressure and stress” (p.16). A recent study combined a scoping review and qualitative interviews with people with dementia to develop a new model of resilience in dementia [9]. The results suggest that a combination of individual, community and societal level resources contribute to resilience in people with dementia, enhancing the wider literature around resilience [7, 8], however the way resilience manifests and is experienced across these resource levels may differ for people with dementia.

Building resilience is one of four key priorities of the European policy framework, Health 2020 [8, 10], and is deemed “essential for modernizing and increasing the performance of health services and public health programmes” [8, p.8]. This is moving on from the historical overuse of deficit-based approaches that ignore peoples’ strengths and assets [11]. This is reflected in national guidance. For example, guidance for implementing the UK Care Act 2014 specifies that “authorities should consider the person’s own strengths and capabilities, and what support might be available from their wider support network or within the community” [3, point 6.63].

While a shift towards strengths-based approaches is evident in international policy, evidence of the benefits of such approaches in practice may not be well-recorded. For example, a key challenge with existing outcome measures for people with dementia is the tendency to focus on deficits, which limits the ability to fully capture the effectiveness of strengths-based approaches, and “the possibility of living well with dementia” [12, p.12]. A recent synthesis of systematic reviews of psychosocial interventions with people with dementia revealed outcome measures assessing intervention impact were mostly deficit-focussed; including cognitive function, mood (anxiety and depression), behaviour (agitation), activities of daily living, and quality of life [13]. Meanwhile, a consultation with people with dementia across Europe revealed that a meaningful psychosocial intervention is one that enhances well-being, confidence, health, social participation, and human rights, with the authors arguing that outcome measures should reflect such positive constructs and strengths [14]. A European working group identified the need to develop new measures that reflect positive functioning, including resilience, as well as to avoid negative terminology within such measures [12].

Current instruments used to measure strengths-based outcomes in people with dementia tend to be developed for other populations and may not be suitable for accurately capturing the experiences of people with dementia. For example, a recent review by Clarke et al., [15] found 35 instruments used to measure well-being in people with dementia, however, only 6 of these had been developed specifically for people with dementia. In relation to measuring resilience, a recent systematic review and psychometric evaluation of resilience measurement scales [16] for people with dementia and their carers found none of the resilience measures used with people living with dementia were developed with or specifically for this population. Of the 51 studies included in the review, only three focussed solely on people with dementia as participants and provided limited evidence of the psychometric properties of the measures in this population. The resilience measures applied in these three studies only focused on individual resources (14-item Resilience Scale, RS-14 [17, 18]; Brief Resilient Coping Scale, BRCS [19]) excluding other important strengths and sources of resilience including social and community engagement [15, 16]. Moreover, the evidence provided from people with dementia in those studies were either focussed on Alzheimer’s Disease or did not disclose specific dementia diagnoses. It is important that measures are developed with input from people living with rarer forms of dementia, as well as more typical, to ensure measures are more inclusive and representative.

*Windle et al*. [16] conclude:

*Further work to establish a new measure may need to consider measuring resilience beyond the individual and include their families and communities as sources of resilience…. People living with dementia and carers should be central to any measurement development or adaptation, in order to embed their lived experiences.* (p.38)

### Aims

In response to the lack of suitable resilience measures specifically for people with dementia, this paper describes the first stage of developing a new measure, the process of developing an item pool. This is an essential stage in measurement development which generates questionnaire items ahead of the last stage of psychometric evaluation where data reduction methods lead to a shorter, final version of the outcome measure. The item pool development builds on initial work which established a new conceptual model of resilience in people with dementia [9], an important first step when considering developing new outcome measures [20]. The item pool reflects the 7 domains presented in the new conceptual model: psychological strengths; practical approaches for adapting to life with dementia; continuing with hobbies, interests and activities; strong relationships with family and friends; peer support and education; participating in community activities; the role of professional support services.

## Methods

The methods used in developing this measure of resilience for people with dementia are informed by those advocated by Streiner et al. [20], and the COSMIN (COnsensus-based Standards for the selection of health Measurement INstruments) group [21]. We also took guidance from the development of the DEMQOL [22], a health-related quality of life measure specifically developed for and validated with people with dementia. The DEMQOL was developed using ‘gold standard’ psychometric methods (as per Streiner & Norman, [23]) and the inclusion of methodological considerations important for people with dementia make this a valuable reference for developing measures specifically for this population.

Streiner et al., [20] advocate for adapting pre-existing measures where possible. Following a mapping exercise, no single pre-existing scale of resilience was deemed suitable for adaptation due to the absence of key themes in the conceptual model of resilience and dementia, e.g., diagnosis acceptance and openness, comparison to others less fortunate, finding comfort in the ordinary, educating oneself about dementia, support from peers [9]. Moreover, other measures of resilience contain items that may not be appropriate when discussing a degenerative condition [16]. For example, questions around ‘bouncing back’ [24, 25], ‘my future feels promising’ [26], and questions around being able to depend on themselves more than others, and being someone others can rely on [27]. For these reasons adaptation would require both addition and deletion of questions. Furthermore, extracting items from several measures would not be feasible because varying response methods and scales across measures would compromise the integrity of the measure. Any amendment such as those described above would constitute a significant adaptation to measures, essentially creating a new measure.

The following stages were implemented in the item generation phase of this work: Phase 1: Developing a ‘long list’ of items: question generation; question and response formatting; content and face validity discussion and initial item reduction. This was undertaken by the core research team, who represented a range of professional disciplines (statistics, clinical psychology, social work and gerontology, with expertise in dementia research, quantitative and qualitative research methods). Phase 2: Cognitive interviews with people with dementia to determine face validity, content validity, and suitability of response format; revising items and final item reduction. Table 1 summarises the methods involved in each step, starting with the development of the conceptual model reported in Windle et al.  [9].

**Table 1:** The steps involved in developing the item pool for a new measure of resilience in dementia

Step	Method
Development of a conceptual model	A scoping review of the literature and qualitative interviews with people with dementia, synthesised into a conceptual model of resilience in dementia. For more information around the development of this conceptual model, see Windle et al. [9].
Question generation	Themes identified in the literature and qualitative interviews used to generate a ‘long list’ of items. Extract quotes from qualitative evidence to inform the development of potential items wherever possible.
Question and response formatting	Discussion among core research team around possible response scales, timeframe and demographics.
Content and face validity discussion, and initial item reduction	Discussion among core research team regarding individual question format and structure. Item list reduced via a process of ranking items and consensus over those deemed most appropriate/least appropriate.
Cognitive interviews for face validity, content validity, and suitability of response format	Interviews with people with dementia (n = 6 people with rare dementias; n = 5 people with more typical forms of dementia). Participants asked to answer each item. Follow-up questions asked by interviewer probing understanding, clarity and usefulness. Participants encouraged to make suggestions for amendment of items where they see fit, and to identify preferred items.
Revising items and final item reduction	Results discussed among core research team. Suggestions for amending questions put forward by interview participants implemented if the core research team agreed that the rewording was clearer and more suitable than the original. Items removed if participants had reported finding difficulty in answering or understanding them. Items with ≤ 1 preference votes also removed.

### Phase 1: developing a ‘long list’ of items

#### Question generation

A new conceptual model of resilience in people with dementia was developed as a starting point to this work [9]. This involved a scoping review of the literature around the personal experience of resilience by people with dementia, as well as qualitative interviews with people living with both typical and rarer forms of dementia by the lead author (A). Thematic analysis of the combined evidence identified themes that were synthesised into a new conceptual model of resilience in dementia (see Windle et al. [9]).

A ‘long list’ of potential resilience measure items were drafted corresponding to each component of the conceptual model of Windle et al. [9], by authors A and B (see Table 2). Quotes or themes from qualitative interviews with people with dementia around their experiences of resilience [9] were adapted into questions where possible. These were put in first-person and present tense (see Table 3 for examples). Neutral or non-directional questions focusing solely on the subject without describing any directionality were also developed and added to the long list. For example, a question about the influence of one’s outlook on life could be directional: ‘I have a positive outlook on life’, or non-directional: ‘my outlook on life helps me manage’. Non-directional questions are phrased so that no assumptions are made about what a person’s outlook is, only that they find it helps them in life. The initial item pool consisted of 140 questions.

**Table 2:** Domains (n = 7) and components (n = 24) from the new conceptual model of resilience in dementia (from Windle et al. [9])

Resilience reserve		
Individual resources		
	Psychological strengths	
		Maintaining sense of humour
		Positivity, gratitude, hope and optimism
		Acceptance of the diagnosis
		Focus on what you can do
		Openness about diagnosis
		Faith or religious beliefs
		Live for the day / in the present
		Comparison to others less fortunate
	Practical approaches for adapting to life with dementia	
		Maintaining pre-diagnosis activity
		Adapting to new lifestyle/changing abilities
		Comfort in the ordinary (e.g. listening to music/TV/coffee)
		Practical adaptation
		Educating oneself / seeking information
	Continuing with hobbies, interests and activities	
		Participating in hobbies and activities
		A sense of purpose
Community resources		
	Strong relationships with family and friends	
		Supportive carer
		Support from family
		Contact with others
	Peer support and education	
		Advocacy and educating others about dementia
		Joining and being part of a group
		Support from peers (living with dementia)
	Participating in community activities	
		Supportive community resources
		Religious activity
Societal resources		
	The role of professional support services	
		Positive connections with healthcare professionals

**Table 3:** Examples of question development from quotes

Example item	Associated quote	Source of quote
A good laugh does me good	A good laugh will do you any good, do you great good	Scoping review: Casey & Murphy (2016)
I take a positive outlook on life	I can’t cope with negative people… it’s got to be a positive outlook on life, so that you can help other people.	Stakeholder engagement activity (The Caban Group)
There are still lots of things I can do	But I mean, once I- I learned to live with it, well, okay, there are lots of things I can’t do. But there are still lots of things that I can do, so… That’s the only way I can look at it	Interviews with people living with a rare dementia and carers
I live for the day	I live for the day, and I don’t look too far into the future… I live for making memories, trying to be useful, and contribute in some way	Stakeholder engagement activity (The CabanGroup)
Making adjustments helps me carry on	I work out, I go to the gym, I still go out with people. I still go to friends. Things have changed, but I still go out…. I’ve had to make adjustments; like I can’t do those things on my own. But I haven’t stopped.	Scoping review: Williamson & Paslawski (2016)
I get tremendous support from my family	I get a lot of support from a lot of people. I get tremendous support from my family.	Stakeholder engagement activity (The Caban Group)
I have a good social life	And- but we’re good- we had a good social life. And we still get a reasonably good social life, so I’ve got nothing to complain about.	Interviews with people living with a rare dementia and carers
I get support from others experiencing similar challenges	We have met people who are of our age-group and at a similar kind of stage in their journey with dementia, shall we say.	Interviews with people living with a rare dementia and carers

#### Question and response formatting

The research team discussed response scales, and following guidance from Streiner et al. [20], a Likert response scale [28] was deemed most appropriate and most user-friendly. It was agreed that various styles of Likert scale would be shown to participants at the cognitive interview stage for feedback on what felt easiest to use. This included varying the number of points on the scale, wording, and use of pictorial aids. Options included a 5-point or a 7-point agreement response scale ranging from “strongly disagree” to “strongly agree”. These included an opt-out option of “no opinion”, “neither agree nor disagree”, “neutral” or “Don’t know”. Pictorial scale options were also explored, such as sad-to-smiling faces, vertical ‘thermometer-style’ visual analogue scales such as those presented in the Canterbury Wellbeing Scales [29], and those presented by Ungar and colleagues (e.g., thumbs down to thumbs up, and glasses ranging from empty to full; [30]).

Retrospective judgements such as “in the last month” may be unnecessarily complex for people who have difficulties with episodic memory [15]. Therefore, it was decided that the most suitable timeframe for responses to questions was “now”. A short demographics section was developed to accompany the resilience measure, to provide context for responses if needed. The demographics section includes information about age, gender, marital status, living arrangements, ethnicity, occupation, education, diagnosis type, and time since diagnosis.

#### Content and face validity discussion, and initial item reduction

The core research team (n = 7) discussed the content and structure of the 140 questions. The team agreed the large number of items in the initial ‘long list’ (n = 140) would result in an unreasonably long cognitive interview with people with dementia. To refine the list to a more manageable length five authors (EB, GW, JS, MPS, ZH) independently assessed each question and ranked them within each component of the model in order of perceived suitability. This was done to ensure coverage of questions across domains and components of the model. The assessment considered how clear, useful, and easy to use each item was. The team agreed that the initial item list would be reduced to a maximum of 70 items. This was to ensure a sufficient number of items to cover components identified in the conceptual model, but not such that it would result in an unreasonably long interview. Where there was a clear preference for some items over others within a cluster, these were retained, and those least favoured (in terms of perceived content validity) were eliminated.

A decision was made to merge ‘supportive care partner’ and ‘support from family’ into one component, due to concerns that ‘partner’ was too specific and may be skipped if a person does not have a partner. A decision was also made to merge ‘joining and being part of a group’ and ‘support from peers living with dementia’, because these were closely related, with joining groups providing opportunities to engage with peers, and there was overlap in the items generated for these themes.

Sixty-two questions were identified from the initial ‘long list’ as suitable for pilot testing in cognitive interviews with some questions (n = 12) reworded to improve readability following discussions within the team. Most amendments were to increase the specificity of items. For example, some items were amended to be more specifically about dementia (n = 6; e.g., “I am open about my condition” became “I am open with other people about my dementia”), and another two questions amended to specifically reflect professional support (e.g. “There are people there to help me if I want it” became “There are health and social care professionals there to help me if I want it”). An additional global resilience question, inspired by Smith et al. [22] who added a global quality of life question in the development of the DEMQOL, was also added to the list for pilot testing bringing the final long list to 63 questions.

### Phase 2: cognitive interviews for face validity, content validity, and suitability of response format

#### Participants for cognitive interviews

Pilot testing of the items was carried out via cognitive interviews with people with dementia. Following recommendations of five to 15 participants for cognitive interviewing in scale development [31], we aimed to recruit a minimum of 10 participants: n = 5 people living with a rare dementia (e.g., Frontotemporal dementia, Posterior cortical atrophy, Primary progressive aphasia, Lewy body dementia, Familial frontotemporal dementia, or Familial Alzheimer’s disease); and n = 5 people living with a more ‘typical’ dementia (Alzheimer’s disease [AD], Vascular dementia [VD], or mixed AD&VD). Participants were recruited via UK-based networks and groups. People living with rare dementias were recruited via the RDS network, with research opportunities advertised through group newsletters and information emails. People living with more typical forms of dementia were recruited via a network of people affected by dementia who are affiliated with the research team at Bangor University.

Informed consent was sought according to the ethically approved RDS Impact study protocol, of which this work is a sub-study [32]. Informed consent was obtained via the online platform GoToMeeting, audio recorded and stored in the secure online portal Data Safe Haven. Participants were recruited if they had capacity to take part, in line with the Mental Capacity Act [33]. Participants were supported to take part via their most suited means. For example, interviews were conducted at the most appropriate time for the participant and could be completed over two sessions if required. Joint interviews were accepted where the person with dementia wished to take part with the support of a partner and both participants had capacity to consent to an interview. An online questionnaire was sent out via Opinio to ascertain demographic information such as gender, date of birth, sexual orientation, marital status, ethnicity, living arrangements, native language, education, occupation, and information regarding the diagnosis of dementia.

#### Procedure for cognitive interviews

Following invitation, participants who expressed interest were contacted by the researcher JR who explained the interview process and arranged the interview. Interviews took place via the online platform GoToMeeting. Participants were asked whether they would be willing to use the camera function for the researcher to ‘share screen’ to display the list item-by-item, both visually and verbally. Interviews were recorded and professionally transcribed by an external company. All data were stored securely in the Data Safe Haven [see 32].

The response scale options and list of questions (n = 63) were sent to participants ahead of the interview to allow participants time to read the questions. To reduce the chance of bias with regards to preference of scale options, questions were listed without a response scale in the pre-interview pack. Interviews were conducted by the lead author who has 8 years’ experience conducting interviews with people with dementia. Participants were consulted about their preferred response options (number of points on the scale, ‘look’ of scale response options, inclusion of a ‘Not applicable’ option, and wording of the ‘middle’ option) at the beginning of the interview. The researcher revisited these options again at the end of the interview to determine whether opinions changed after completing the list of items.

A basic five point black and white Likert scale was added to questions for the interview to facilitate discussion and answering of questions. Item order was altered between participants to reduce any order effects and effects of fatigue on results. Items corresponding to each component of the model remained in ‘clusters’ as alteration of item order took place, for ease of exploring preferences between items similar in meaning or wording. Following methods used by Smith et al. [22] in the development of the DEMQOL, participants were then asked to answer each question, with follow-up questions asked to ascertain whether the question had been understood as intended (e.g., ask why participants answered the way they did). They were also asked to vote if they had a preference for one item over the others within a cluster, (items that are similar in meaning). This approach differed slightly to the method advocated by Streiner et al. [20] of asking participants to rephrase questions, because it was considered too complex a task to use for some people with dementia [22]. Moreover, participants were encouraged to make suggestions for amending items they felt could be better worded, and to identify items that they did not like or understand. Participants also explored whether they felt any item within a ‘cluster’ (i.e., corresponding to the same component in the model) was better or easier to understand and answer than others.

## Results

Ten participants took part in cognitive interviews (n = 5 people with a rare dementia, n = 5 people with more typical forms of dementia). One additional participant with limited verbal output, as a consequence of living with progressive non-fluent aphasia, was supported to participate by writing responses on a printed document with written instructions. Three further people showed interest in taking part but were deemed to lack capacity. Time spent with participants during cognitive interviews ranged from 70 to 145 min (M = 110.3; SD = 22.8 min), although it should be noted the 145-minute interview was conducted on two separate days (90 min and 55 min each). Table 4 displays key demographic information of participants. Participants (male n = 6, female n = 5; mean age = 69.36) had been diagnosed with dementia for a varying number of years (M = 4.8 years; SD = 3.12; range 1–9 years), and also reported living with a range of confirmed diagnoses (please see Table 4).

**Table 4:** Demographic information of cognitive interview participants

Characteristic		Participants (n)
Gender		
	Male	6
	Female	5
Age		
	Mean	69.36 years
	Range	55–80 years
Marital status		
	Married	9
	Divorced/separated	2
Lives with		
	Spouse	9
	Alone	2
Ethnicity		
	English/Welsh/Scottish/Northern Irish/British	9
	Chinese	1
	Syrian and Greek	1
Employment		
	Employed or self-employed	1
	Retired	10
Diagnosis of dementia		
	Progressive nonfluent aphasia	2
	Primary progressive aphasia	2
	Posterior cortical atrophy	1
	Logopenic progressive aphasia	1
	Alzheimer’s disease & Vascular dementia	2
	Alzheimer’s disease	3
Time since diagnosis		
	Mean	4.8 years
	Range	1–9 years

There was a mixed response to including a N/A (not applicable) option. Pre-interview participants were generally in favour of including this option, but post-interview some (n = 2) felt it was not actually needed, whereas another felt it would have been useful for some questions because “If you’re in your 90s you might not have any friends left”. The research team agreed to include a N/A option in the next stage of scale development, due to the overall favourable opinion of participants towards it.

The various scale formatting options presented were often considered too difficult or confusing, and accessibility issues were raised (e.g., visual challenges affecting ability to differentiate red from green in coloured scales). These feelings tended not to change when revisiting these options at the end of the interview. Suggestions were to keep the scale simple, with tick boxes that people are familiar with. A strong preference (n = 7) was evident for including 5 points on the scale. Whilst, some (n = 3) felt the word ‘strongly’ was not needed, because to agree/disagree alone would be sufficient, the research team decided to keep the ‘strongly’ options at either end of the scale due to the strong preference for a 5-point scale. Therefore, the final response scale will be a simple tick-box 5-point Likert scale, ranging from ‘strongly disagree – strongly agree’.

There was variation in preference for the ‘middle option’ wording. Both ‘neither agree nor disagree’ and ‘I don’t know’ received the highest preference votes (n = 5 each; Note: some participants voted for more than one option). However, ‘neither agree nor disagree’ received no negative feedback and was generally familiar to participants. The research team therefore considered this wording most appropriate.

A debate around language was raised regarding the use of the word ‘dementia’ within items. Some (n = 3) suggested using alternative wording (e.g., ‘condition’) because they did not like or use the word dementia, whereas others (n = 6) felt it important to use (one person had no opinion on this). Given overall group consensus for inclusion of the word ‘dementia’, this will be kept within items going forward. Further details of scale formatting discussions and decisions, and constructive feedback from people with dementia are presented in Appendix A (Electronic Supplementary Material).

### Revising items and final item reduction

The results of the cognitive interviews informed the revision and deletion of items. Suggestions made by participants for amending items were discussed among the core research team and implemented where there was agreement of improvement. All queries or comments raised by participants were considered by the team. Finally, the Question Understanding Aid (QUAID) tool (http://quaid.cohmetrix.com) was consulted to identify potentially problematic terms and phrases within items. Items were amended or new items added if the core team agreed they could be made more clear or concise.

Exclusion criteria for the final item pool was as follows (in order of priority): Items deemed difficult to understand or answer by any participant, or low preference items (items with ≤ 1 preference votes from participants) within each ‘cluster’ were removed. In addition, where there was a strong preference item (≥ 4 votes higher than other items within a cluster), the other items within the cluster were removed.

The main interview findings regarding the process of item selection, removal, and amendment can be found in Appendix B (Electronic Supplementary Material). Eighteen items were removed for difficulty of understanding (n = 7) or difficulty answering (n = 11). Items removed for difficulty of understanding or answering were generally spread across components and domains (18 items across 13 components). However, all items relating to one component, ‘maintaining pre-diagnosis activity’, were difficult for multiple participants and a decision was made to remove the component and its corresponding items from the final list. Other items were removed due to low preference (votes ≤ 1: n = 6), and presence of a preferred item within component cluster (n = 4). Moreover, items were amended to enhance clarity or conciseness based on suggestions of participants (n = 10), QUAID (n = 7), and both participants and QUAID (n = 2). Two new items were also added: One was added due to varied interpretations of its predecessor in the initial list, and another was added after a decision was made to separate the merged ‘family’ and ‘partner’ components and related questions, so that corresponding items were available for both. The resulting final item pool list includes 37 questions for further investigation (see Appendix C, Electronic Supplementary Material) The 37-item pool list includes 13 items corresponding to the psychological strengths domain; 10 within practical approaches for adapting to life with dementia; 2 in continuing with hobbies, interests and activities; 6 in strong relationships with family and friends; 3 in peer support and education; 1 in participating in community activities; and 2 in the role of professional support services.

## Discussion

This paper describes the first stages in the development of a new measure of resilience for people living with dementia. This new measure is being developed in response to an absence of instruments measuring strengths-based outcomes specifically in people with dementia, and in particular, a lack of suitable resilience measures for people with dementia [16]. As international policy shifts towards advocating strengths-based approaches to practice, it is important that outcome measures reflect this shift, acknowledging that there is more to consider than historical deficit-based approaches that have prevailed in relation to dementia.

Existing measures of resilience used with people with dementia have predominantly focussed on individual resources alone, and none have been developed specifically for and with people living with dementia [16]. Conversely, this new measure is being developed together with people living with dementia, and includes items that are informed by the individual, community and societal level resources identified in accounts by people living with dementia of their own experiences of resilience. Following previous work to develop a new conceptual model of resilience in people with dementia [9], this paper reports the process of developing an item pool for a new measure of resilience specifically for people with dementia.

Led by the experiences and important observations of people living with dementia, items corresponding to seven domains of resilience (and components within) are presented. Cognitive interviews reduced the number of items from 63 to 37, with criteria for item removal shaping the final list. Whilst the components of the domains presented in the final item pool list largely map on to the conceptual model [9], preliminary field testing and exploratory factor analysis may change the domains and components within, or elicit new ones, in the final measure.

This work has several strengths. Following gold standard procedures advocated by Streiner et al. [20] and COSMIN quality standards for study design [21] ensured rigour of the process. The initial set of items were derived from themes identified during interviews with people living with dementia, using direct quotes where possible. A team of professionals with expertise in a range of disciplines relating to dementia research and to resilience were involved in all aspects of the process, coming together to discuss and gain consensus on any issues that arose. Moreover, cognitive interviews with people living with dementia explored the initial set of items, with items removed or amended according to participant feedback, demonstrating the extensive attention paid to content and face validity of the resulting item pool.

The final version is intended to be a self-reported outcome measure (i.e., completed by the person themselves (either alone, or together with someone supporting them) meaning it will be appropriate for people living with mild to moderate dementia. Other research confirms that people in the milder to moderate stages of the condition are capable of providing reliable responses on widely used outcome measures [e.g., 22, 34]. For those with more severe dementia, proxy measures which enable another person to provide responses on behalf of a person living with dementia are often utilised. Following the final psychometric validation of this measure, this work could be extended to develop a proxy measure of resilience to capture the resilience of those with more advanced dementias.

A strength of the current sample is the inclusion of people living with a range of dementia diagnoses within the sample. The experiences of people living with rarer forms of dementia are often overlooked in dementia research [e.g., 35]. However, whilst inclusion of rarer dementias is a strength, we acknowledge that it was not possible to include all types of dementia, and the experiences of those living with the rarest types may not be represented.

Another strength of the sample is that an equivalent number of male and female participants, falling within a wide age range and a range of years post-diagnosis, took part. However, a limitation of this work is a lack of ethnic diversity within the sample. Most participants were Caucasian and British, with only two participants coming from other backgrounds. This lack of ethnic diversity is common in dementia research [e.g., 36].

### Implications for future research

The ‘long’ list of resilience questions generated by this study provide the basis for further, larger psychometric studies to enable a final and shorter resilience outcome measure for use by research, policy, and practice.

The next stage of this research should strive to extend sample diversity and find ways to reach out to people from other ethnic backgrounds, such as via community-based outreach, as suggested by Brijnath et al. [37]. It will be important that any translation of the measure into different languages and different cultures follows standardised approaches, including forward-back translation and cross-cultural validation with the involvement of people from the intended target culture, to ensure ambiguities in meaning are addressed.

The next stages of psychometric evaluation should continue to apply robust methodology [20, 21], to ensure the resulting outcome measure of resilience for people living with dementia is of gold-standard quality. This will allow researchers and professionals to add a strengths-based measure to their repertoire; a tool likely to be valuable in assessing positive responses to services and interventions for people living with dementia.

## Conclusions

As international policy shifts towards promoting strengths-based approaches to practice, outcome measures should also reflect this shift, moving away from historical deficit-based approaches that have prevailed in relation to dementia. The process described in this paper incorporates experiences and expertise from people with dementia in relation to their own resilience. The resulting set of items towards a new measure of resilience was therefore understandable, acceptable, and accessible to participants. The final item pool consisted of 37 items, to be used in the next phase of measure development: Preliminary field testing.

### Electronic supplementary material

Below is the link to the electronic supplementary material.

Supplementary Material 1

Supplementary Material 2

Supplementary Material 3

## Data Availability

The datasets generated and analysed during the current study are available from the corresponding author on reasonable request.

## List of abbreviations

AD: Alzheimer’s disease
BRCS: Brief Resilient Coping Scale
COSMIN: COnsensus-based Standards for the selection of health Measurement Instruments
DEMQOL: Dementia Quality of Life measure
M: Mean
N/A: Not applicable
QUAID: Question Understanding Aid
RDS: Rare Dementia Support
RS-14: Resilience Scale
SD: Standard Deviation
UK: United Kingdom
VD: Vascular dementia
