# Supplementary material for: Development of an item pool for a patient reported outcome measure of resilience for people living with dementia
Source: J Patient Rep Outcomes. 2023 Sep 27;7:96. doi: 10.1186/s41687-023-00638-z (PMC10533765; doi:10.1186/s41687-023-00638-z)
Supplement: Supplementary file 1 — Supplementary Material 1 [file 41687_2023_638_MOESM1_ESM.pdf]

Appendix A. Response scale formatting discussions and decisions.

Discussion		Important observations by people with dementia	Outcome
**Scale formatting**			
	Including an N/A option	"I think it's useful to have a not applicable option"<br>"Yes you need something like that don't you because if it doesn't apply to you, what do you do?"<br>"The idea of the N/A, I think probably-probably you need something like that"<br>"I don't think so, because that already comes up in 'neither agree nor disagree'"<br>"Neither agree nor disagree would cover that"	Decision by core team to include for preliminary field test to determine its usefulness.
	Colour dots (red -> green)	"That's exactly what you'd expect…I thought that was rather nice you know, I was expecting normal-normal, you know, black [dots]"<br>"I'm used to that, I quite like that"<br>"Yeah, that's workable. But there isn't a 'neither agree nor disagree', which I'm used to"<br>"The coloured dots wouldn't work with a lot of people, because a lot of people, like me, are partly colourblind"<br>"I do not like it"	Do not use: Feedback that those with vision problems such as colour-blindness would find this difficult. Decision to amend, see "Additional formatting suggestions" below.
	Smiley faces (sad to smiling scale)	"I quite like the smiley faces"<br>"I like this one"<br>"I don't need smiley faces or glasses"<br>"I don't like the smileys"<br>"I'm not comfortable with doing this... I don't see so well little things like that...they're all looking similar so I have a problem deciding which is which"<br>"When it came to smiling faces I was like, oh, I don't know what that means"	Do not use: liked by some, but others found it confusing.
	Water Glasses (empty to full)	"I haven't got any problems with that one"<br>"I understand this but it's not my favourite"<br>"I personally wouldn't use that one"<br>"I don't like that at all"<br>"I don't like that one at all"<br>"That just messes with your head"<br>"I do not like it very much"	Do not use: Generally disliked.

Thermometer (vertical line)	"I do think the thermometer is useful, 'cause it give you more scope between strongly disagree and strongly agree … it might be too difficult, complicated for some people"<br>"I guess it can be more precise"<br>"I don't like that at all, that's a bit too complicated for people with dementia...I wouldn't use that at all"<br>"That's very difficult...I just can’t work it out really"<br>"My brain has got to work hard to see this one"<br>"I think it's a bit complicated"<br>"No, I didn't like that"	Do not use: complicated and difficult to use.
Thumbs (down, mid-way, up: 3-points in example)	"What I like actually, is the thumbs down, thumbs up. And the reason I like that is…when you're getting advanced with your dementia, you're going back to your childhood and when you're a child you learn the thumbs up and thumb down don't you"<br>"I would go simple and I would do a thumb, because if you give too many options it blurs it, that's very personal, cause i can struggle with things like that"<br>"The thumbs didn’t give me enough options"<br>"Come on, we can do better than that"<br>"A lot of people wouldn't understand that" [translated from Welsh]<br>"There's not enough range and it’s also confusing because of the middle one"<br>"The middle picture, I've never seen that before...I don't know what else you'd put there to be honest...That's better for people who may be struggling with communication... don't understand the middle one"<br>"It's that middle one... mind you you'll have the writing as well won’t you...yeah that's ok"<br>"That one is more straightforward but I never use it so, no...It doesn't feel as if its gathering and much information as the others"<br>"I don't much like it"	Do not use: confusing and lack of range.
Additional formatting suggestions	"Just a square box would do...so they can put a cross or a tick in it"<br>"If you want to do colours then fine, but I don’t need colours"<br>"Keep it simple [referring to the possibility of including three options and boxes to tick]"<br>"One project i did take part in and they used just red, yellow and green... and it was red, no; yellow, don't know; green, yes...it's got the colours that you know"<br>"Tick the boxes...but then if somebody has trouble with their reading...you've got to be inclusive haven't you"<br>"I think you use something like a cross in a box don't you...you just tick or cross that box" [feels like the easiest way to do it]	Use a simple scale: Based on feedback of the above more elaborate examples, and suggestions to keep the scale simple, a decision was made by the group for a tick-the-box type scale.

**Wording of the 'Middle option'**		
"Neither agree or disagree"	<u>5 votes (1 was second choice)</u><br>"I personally like 'neither agree nor disagree'"<br>"I agree with that"<br>"I'm used to it"	Decision by research team to use: An option that is already familiar and acceptable.
"Neutral"	<u>4 votes (2 were second choice)</u><br>"'Neutral' I can understand as I'm not quite sure if I'm disagree or agree"<br>"Neutral, I have difficulty using that...It doesn't really compute with me very well"	Do not use: The above option was preferred, and one person found 'neutral' confusing.
"I don't know"	<u>5 votes</u><br>"I don't know almost means N/A"<br>"I don't know sounds good"	Do not use: Decision made to include 'neither agree nor disagree' as middle option because it received no negative feedback and was familiar.
"No Opinion"	<u>2 votes</u><br>"I really like the 'no opinion'"<br>"I don't like the idea of 'no opinion'"<br>"No opinion makes it just sound like you've got no interest"	Do not use: Lack of preference and particularly disliked by some people with dementia.
**Number of points on a scale**		
Three-point scale	<u>3 votes</u><br>"If you disagree, what's the point in saying I strongly disagree? If you've disagreed that's the end of it isn't it"<br>"Three is ample"<br>"Disagree, neutral, agree"<br>"I think that's not enough, because you could perhaps agree, but not strongly agree"	Do not use: Low preference votes and lack of range (see also feedback for 'thumbs' above)
Five-point scale	<u>7 votes</u><br>"I think you need 5"<br>"I think less is better and I like the five…I think you get better feedback with the five"<br>"Five point scale is enough"	Decision to use as a 5-point scale due to strong preference.
Seven-point scale	<u>1 vote</u><br>"Too difficult"<br>"The seven point scale would give me more options, but as a collective investigation, it could be confusing…my friends [with dementia] would definitely have a problem with it"	Do not use: Low preference and deemed too difficult.

**Additional comments**		
Additional comments: Use of word 'strongly'	"I don’t think there's a need really for the 'strongly' part of it"<br>"Do we really need the 'strongly disagree'? Wouldn't 'disagree' do?"<br>"I still don’t think we need the word 'strongly', because what does it mean 'strongly disagree', strongly agree'? You either agree or you don't"	The group consensus was to have a 5-point scale. Therefore the research team made the decision to keep 'strongly' at either end of the scale for clarity.
