# Supplementary material for: Development of an item pool for a patient reported outcome measure of resilience for people living with dementia
Source: J Patient Rep Outcomes. 2023 Sep 27;7:96. doi: 10.1186/s41687-023-00638-z (PMC10533765; doi:10.1186/s41687-023-00638-z)
Supplement: Supplementary file 2 — Supplementary Material 2 [file 41687_2023_638_MOESM2_ESM.pdf]

Appendix B. Item selection, removal, and amendment.

Domain	Component	Questionnaire item	Important observations	Outcome
**Individual resources**				
	Psychological strengths			
	Maintaining sense of humour	1. A good laugh does me good		Keep: Q1
		2. I have a good sense of humour	Q2: Difficulty answering (PLWD): Varied interpretations, and a suggestion that someone else would be able to answer this question on your behalf. Concern interpretation of question was not reflecting *maintaining sense of humour* aspect of resilience - new alternative question added.	Remove: Q2<br>Add: I can see the funny side of things
		3. I like to laugh	Q3: Low preference (votes ≤ 1)	Remove: Q3
	Positivity, gratitude, hope and optimism	4. My outlook on life helps me manage	Q4: Difficulty understanding (PLWD): "What does that even mean?"<br>Q4: Suggestion that 'outlook' items are more difficult to understand.	Remove: Q4
		5. My approach to life helps me manage	Q5: Strong preference for Q8 within this cluster.	Remove: Q5
		6. My attitude to life helps me manage	Q6: Strong preference for Q8 within this cluster.	Remove: Q6
		7. I take a positive outlook on life	Q7: Suggestion that 'outlook' items are more difficult to understand.	Remove: Q7
		8. Taking a positive attitude helps me manage	Q8: There was a strong preference for this item, and for using the word 'positive' ($\geq$4 votes higher than other items within cluster).	Keep: Q8
	Acceptance of the diagnosis	9. I've accepted my diagnosis	Q9: Suggestion for clarity (QUAID): I've -> I have	Amend: Q9: I have accepted my diagnosis
		10. I accept my condition		Keep: Q10

	11. I make the best of things	Q11: Suggestion for clarity (QUAID): I make the best of my situation	Amend: Q11: I make the best of my situation
Focus on what you can do	12. I know my strengths and limitations	Q12: Difficulty answering (PLWD): "I haven't a clue because you'd have to sort of think about what you mean by that... I think that really is a difficult question."	Remove: Q12
	13. There are still lots of things I can do		Keep: Q13
	14. I do the best I can		Keep: Q14
Openness about diagnosis	15. I don't have to hide my diagnosis from anybody	Q15: Low preference (votes ≤ 1)	Remove: Q15
	16. I am open with other people about my dementia	Q16: There was a strong preference for this item (≥4 votes higher than other items within cluster)	Keep: Q16
	17. I tell others about my dementia	Q17: Low preference (votes ≤ 1)	Remove: Q17
Faith or religious beliefs	18. My faith/religion/beliefs help me manage.	Q18: Suggestion for clarity: "My personal beliefs help me manage (e.g. faith, religion, spirituality)".<br>Q18: Suggestion to re-word (PLWD): remove "manage"<br>Q18: Suggestion for clarity (QUAID): Spirituality -> spiritual beliefs.	Amend: Q18: My personal beliefs help me live with my dementia (For example: faith, religion, spiritual beliefs)
Live for the day/in the present	19. I live for the day	Q19: Difficulty answering (PLWD):"It doesn't really have any meaning. I have to live every day."<br>Q19: Low preference (votes ≤ 1)	Remove: Q19
	20. I take each day as it comes	Q20: There was a strong preference for this item (≥4 votes higher than other items within cluster)	Keep: Q20
	21. I don't worry about tomorrow	Q21: Difficulty answering (PLWD): Varied interpretations: Literal tomorrow vs looking into the future.<br>Q21: Low preference (votes ≤ 1)	Remove: Q21
Comparison to others less fortunate	22. I appreciate what I have	Q22: Difficulty answering (PLWD): "I don't know what to say to that" and "I have to think about this one."	Remove: Q22

	23. There are lots of people a lot worse off than me	Q23: Suggestion for conciseness (PLWD): remove 'a lot'	Amend: Q23: There are lots of people worse off than me
	24. There’s always someone else worse off than me		Keep: Q24
Practical approaches for adapting to life with dementia			
Maintaining pre-diagnosis activity	25. I keep doing what I’ve always done	Q25-27: Difficulty understanding (PLWD): Multiple participants reported not understanding the questions. Some said they now do more, which also leads to difficulty answering. "What does it matter what I did 10 years ago?" "I don't think anyone can keep up the activities they did before dementia" "These questions could make you feel a bit negative, not as confident"	Remove: Q25
	26. I carry on doing the things I did before		Remove: Q26
	27. I keep up the activities that I did before dementia		Remove: Q27
	28. I keep up my interests	Q28: Lack of clarity (PLWD). Interpreted in the context of 'now' rather than pre-diagnosis activity.	Amend: Q28: "I keep up my activities and interests" due to how it is being interpreted, to fall within Participating in hobbies and activities cluster.
Adapting to new lifestyle/changing abilities	29. Making some changes helps me carry on with my life	Q29: Lack of clarity (PLWD): "It doesn’t say why I’ve got to carry on. This doesn’t specify dementia"	Amend: Q29: Making changes helps me live with my dementia
	30. I am adapting to the changes from living with dementia	Q30: Low preference (votes ≤ 1)	Remove: Q30
	31. I am adapting to living with dementia		Keep: Q31
Comfort in the ordinary (e.g. listening to music/TV/coffee)	32. I find enjoyment in everyday things		Keep: Q32
	33. I find enjoyment in the little things	Q33: Suggestion for clarity (PLWD): "I find enjoyment in the more simple things"	Amend: Q33: I find enjoyment in the simple things in life

Practical adaptation	34. I make practical adaptations to make life easier	Q34: Suggestion for clarity (QUAID): 'adaptations' may be too technical	Amend: Q34: I have made practical changes to make my life easier
	35. When I am faced with a problem, I work to find a way around it	Q35: Suggestion for clarity (QUAID): 'work' may be too vague.	Amend: Q35: When I am faced with a problem, I find a way around it
	36. I try to find ways around problems that arise	Q36: Suggestion for clarity (QUAID): 'try' and 'arise' may be too vague.	Amend: Q36: I find ways around problems in my life
Educating oneself / seeking information	37. I find information that helps me live with dementia		Keep: Q37
	38. I learn as much as I can about dementia	Q38: Suggestion for clarity (PLWD): “I learn as much as I can about dementia to help me live with it”	Amend: Q38: Learning about dementia has helped me to live with it

Continuing with hobbies, interests and activities

Participating in hobbies and activities	39. I have hobbies	Q39: Low preference (votes ≤ 1)	Remove: Q39
	40. I have interests	Q40: Strong preference for Q41 within this cluster.	Remove: Q40
	41. I keep up my hobbies and interests	Q41: There was a strong preference for this item ($\geq$4 votes higher than other items within cluster).	Keep: Q41
A sense of purpose	42. I have a purpose in life	Q42: Low preference (votes ≤ 1)	Remove: Q42
	43. I do things that are meaningful to me	Q43: Difficulty understanding (PLWD): "I don’t know, that’s difficult". "What does that mean?"	Remove: Q43
	44. I am able to do things important to me	Q44: Suggestion for clarity (PLWD): "I do things important to me", because "am able to" made participants think about what they were unable to do.	Amend: Q44: I do things important to me

Community resources

Strong relationships with family and friends

Supportive care partner & Support from family (component themes merged)	45. I am happy with the support I receive from my family/partner	Q45: Suggestion for clarity (PLWD): Several participants said that 'family' and 'partner' should be two separate questions.	Amend: Q45: I am happy with the support I receive from my family
	46. I am happy with the support I receive from my family (including partner)	Q46: Suggestion for clarity (PLWD): Several participants said that 'family' and 'partner' should be two separate questions.	Amend: Q46: I am happy with the support I receive from my partner
	47. I get tremendous support from my family/partner	Q47: Difficulty answering (PLWD): Suggestion that 'tremendous' was too strongly worded	Remove: Q47
	48. My family is supportive		Keep: Q48. Add new item: My partner is supportive
Contact with others	49. I am happy with the support I receive from my friends	Q49: Difficulty answering (PLWD): "That's difficult...I could give that a 1 or a 4, because I only *receive* support if I see any of them"	Remove: Q49
	50. I feel supported by my friends		Keep: Q50
	51. I get support from a lot of people	Q51: Difficulty answering (PLWD): "What does 'a lot' mean?"	Remove: Q51
	52. I have a good social life	Q52: Suggestion to re-word (PLWD): "My social life is satisfying"	Amend: Q52: My social life is satisfying
Peer support and education			
Advocacy and educating others about dementia	53. Talking about dementia to others is important	Q53: Difficulty understanding (PLWD): Suggestions by PLWD indicate misunderstanding: "Being able to talk to others about my condition is important." & "Talking to others in the same position as me is helpful and important."	Amend: Q53: Educating other people about my dementia is important
Joining and being part of a group & Support from peers living with dementia	54. Meeting others going through difficulty has shown me I'm not on my own	Q54: Suggestion for clarity (QUAID): 'others' may be too vague	Amend: Q54: Meeting other people going through difficulty has shown me I am not on my own

(component themes merged)	55. It helps to meet people who don't judge me	Q55: Difficulty understanding (misunderstanding) (PLWD): Participants attribute to meeting other people in general, comments such as "Wouldn't that be nice!". Q55: Low preference (votes ≤ 1)	Remove: Q55
	56. I get support from others experiencing similar challenges	Q56: Suggestion for clarity (QUAID): 'others' may be too vague	Amend: Q56: I get support from other people experiencing similar challenges
Participating in community activities			
Supportive community resources	57. I am satisfied with the support I receive from my community (e.g., people in my neighbourhood, clubs and societies).	Q57: Strong preference for Q58 within this cluster.	Remove: Q57
	58. I am part of a supportive community (e.g., people in my neighbourhood, clubs and societies).	Q58: Strong preference for this item (≥4 votes higher than other items within cluster) Q58: Lack of clarity (PLWD): Participants tended to focus on the 'neighbourhood' part of examples list and interpret as being 'local' only. Q58: Working memory overload (QUAID): due to length and complexity of examples list.	Amend: Q58: Examples amended to "For example: online or face to face groups, forums, clubs and societies"
**Societal resources**			
The role of professional support services			
Positive connections with healthcare professionals	59. The support I receive from health and social care professionals meets my needs		Keep: Q59
	60. I am happy with the support I receive from health and social care professionals.		Keep: Q60
	61. There are health and social care professionals there to help me if I want it	Q61: Difficulty answering (PLWD): "It's not easy but if you fight for it, it's there. It's a difficult question to answer in that respect…."	Remove: Q61

	62. There is professional support for me if I need it	Q62: Difficulty answering (PLWD): "I don’t know what support there is."	Remove: Q62
Additional global resilience question	63. I am a resilient person	Q63: Difficulty answering: "I don’t know. Feels very hard to answer...I judge myself harshly in most things." Comments that this question might be better answered by somebody else, rather than the person themselves.	Remove: Q63

Note: PLWD – people/person with dementia; QUAID – Question Understanding Aid Tool.
