# Supplementary material for: Development of an item pool for a patient reported outcome measure of resilience for people living with dementia
Source: J Patient Rep Outcomes. 2023 Sep 27;7:96. doi: 10.1186/s41687-023-00638-z (PMC10533765; doi:10.1186/s41687-023-00638-z)
Supplement: Supplementary file 3 — Supplementary Material 3 [file 41687_2023_638_MOESM3_ESM.pdf]

Appendix C. The final item pool.

1. A good laugh does me good
2. I can see the funny side of things
3. Taking a positive attitude helps me manage
4. I have accepted my diagnosis
5. I accept my condition
6. I make the best of my situation
7. There are still lots of things I can do
8. I do the best I can
9. I am open with other people about my dementia
10. I take each day as it comes
11. There are lots of people worse off than me
12. There is always someone else worse off than me
13. Making changes helps me live with my dementia
14. I am adapting to living with dementia
15. I find enjoyment in everyday things
16. I find enjoyment in the simple things in life
17. I have made practical changes to make my life easier
18. When I am faced with a challenge, I find a way around it
19. I find ways around problems in my life
20. I find information that helps me live with dementia
21. Learning about dementia has helped me to live with it

22. I keep up my activities and interests
23. I keep up my hobbies and interests
24. I do things important to me
25. I am happy with the support I receive from my family
26. My family is supportive
27. I am happy with the support I receive from my partner
28. My partner is supportive
29. I feel supported by my friends
30. My social life is satisfying
31. Educating other people about my dementia is important
32. Meeting other people going through difficulty has shown me I am not on my own
33. I get support from other people experiencing similar challenges
34. I am part of a supportive community (For example: online or face to face groups, forums, clubs and societies)
35. My personal beliefs help me live with my dementia (For example: faith, religion, spiritual beliefs)
36. The support I receive from health and social care professionals meets my needs
37. I am happy with the support I receive from health and social care professionals.
